# Esophageal *Candida* Infection and Esophageal Cancer Risk in Patients With Achalasia

**DOI:** 10.1001/jamanetworkopen.2024.54685

**Published:** 2025-01-14

**Authors:** Xiaopei Guo, Suk Yee Lam, Vincent T. Janmaat, Pieter Jan F. de Jonge, Bettina E. Hansen, Ivonne Leeuwenburgh, Maikel P. Peppelenbosch, Manon C. W. Spaander, Gwenny M. Fuhler

**Affiliations:** 1 Department of Gastroenterology and Hepatology, Erasmus University Medical Center, Rotterdam, the Netherlands; 2 Department of Epidemiology & Biostatistics, Erasmus University Medical Center, Rotterdam, the Netherlands; 3 Department of Gastroenterology and Hepatology, Franciscus, Gasthuis en Vlietland, Rotterdam, the Netherlands

## Abstract

**Importance:**

Patients with achalasia face a higher risk of developing esophageal cancer (EC), but the surveillance strategies for these patients remain controversial due to the long disease duration and the lack of identified risk factors.

**Objective:**

To investigate the prevalence of esophageal *Candida* infection among patients with achalasia and to assess the association of *Candida* infection with EC risk within this population.

**Design, Setting, and Participants:**

This retrospective cohort study included patients with achalasia diagnosed at or referred for treatment and monitoring to the Erasmus University Medical Center in Rotterdam, the Netherlands, between January 1, 1980, and May 31, 2024. Data analysis was conducted from August 1 to October 31, 2024.

**Exposure:**

Esophageal *Candida* infection.

**Main Outcomes and Measures:**

The primary outcomes were the prevalence of esophageal *Candida* infection and its association with EC development among patients with achalasia. Associations were estimated using time-dependent Cox proportional hazards regression models with esophageal *Candida* infection as a time-varying covariate, adjusting for age at diagnosis and sex.

**Results:**

This study included 234 patients with achalasia (median [IQR] age at diagnosis, 45 [32-63] years; 117 [50%] male), with a median follow-up time of 13 (4-22) years. Esophageal *Candida* infection was identified in 29 patients (12%), while EC was observed in 24 patients (10%). Esophageal cancer risk analysis was performed for 207 patients with 2 or more consecutive endoscopy follow-up visits (median [IQR] age at diagnosis, 43 [32-60] years; 104 [50%] male). The median (IQR) follow-up time for this subgroup was 16 (9-26) years. Among these patients, esophageal *Candida* infection was independently associated with an increased risk of EC (adjusted hazard ratio [AHR], 8.24 [95% CI, 2.97-22.89]). Additionally, age at diagnosis (AHR, 1.06 [95% CI, 1.03-1.10]) and male sex (AHR, 3.34 [95% CI, 1.08-10.36]) were independently associated with EC risk.

**Conclusions and Relevance:**

This retrospective cohort study found that prior esophageal *Candida* infection, older age at diagnosis, and male sex were associated with increased risk of EC among patients with achalasia. These findings provide an important rationale for optimizing the monitoring of patients with achalasia.

## Key Points

**Question:**

Is esophageal *Candida* infection associated with increased risk of esophageal cancer among patients with achalasia?

**Findings:**

In this retrospective cohort study of 234 patients with achalasia, esophageal candidiasis was identified in 12% of patients. Prior esophageal *Candida* infection was associated with a higher risk of esophageal cancer development in these patients.

**Meaning:**

These findings highlight a need for improved reporting on esophageal *Candida* infection during monitoring of patients with achalasia, and regular surveillance endoscopy should be considered for patients with achalasia and *Candida* infection.

## Teaser

This cohort study investigates the prevalence of esophageal *Candida* infection and the association of *Candida* infection with esophageal cancer risk among patients with achalasia.

## Introduction

Achalasia is an uncommon esophageal motility disorder with an estimated prevalence of 6.5 to 15.7 per 100 000 individuals.^1,2^ Loss of esophageal peristalsis and a failed relaxation of the lower esophageal sphincter caused by an autoimmune reaction or virus infection lead to an impaired flow of ingested food to the stomach in patients with achalasia, resulting in food stasis. Consequently, patients may experience symptoms such as dysphagia, regurgitation of undigested food, chest pain, heartburn, and weight loss. Because of the unknown etiology for achalasia, current treatments primarily focus on alleviating symptoms rather than targeting the specific causes of the disease.

Patients with achalasia face an increased risk of developing esophageal cancer (EC), in particular esophageal squamous cell carcinoma (ESCC).^3,4^ While the annual incidence is disputed,^1,5,6^ recent meta-analyses suggest an EC incidence rate of 3.3 cases per 1000 patients with achalasia, which exceeds the incidence rate in the general population at least 10-fold.^7,8^ Treatments for achalasia, such as pneumatic dilation, laparoscopic Heller myotomy, and peroral endoscopic myotomy, can improve the esophageal transit by reducing the resting pressure of the lower esophageal sphincter. Nevertheless, patients may still experience ongoing or recurrent esophageal distention and retention of food, and increased risk of EC persists even after treatment.^2,9^

Despite the strong correlation between achalasia and EC, there are only a few studies focusing on the pathogenesis of neoplastic transformation in this disease, which may differ from that of sporadic EC. One of the hypotheses is that long-term food stasis promotes bacterial overgrowth and fermentation, and the persistent chemical irritation causes chronic inflammation and dysplasia of the esophageal mucosa^10,11,12^ and exacerbates the ongoing inflammation caused by the autoimmune reaction. A role for the microbiome in initiation or progression of cancer is becoming ever clearer.^13^ Besides *Helicobacter pylori* as a main driver of gastric carcinogenesis, bacterial contributions to diverse gastrointestinal cancers have now been identified.^14^ Bacterial toxins, metabolites, and infection-induced inflammation have been suggested as drivers of carcinogenesis. In addition, fungal infections may also drive inflammatory processes and promote cancer development, although the role of the mycobiome in cancer is only slowly being elucidated. Several recent studies have pointed toward an altered mycobiome in diverse cancerous tissues.^15,16^ A noteworthy candidate is the yeast of the genus *Candida,* which colonizes the gastrointestinal mucosa and can cause opportunistic infections, including esophagitis. Epidemiological and pathological studies have underscored the ability of *Candida* species infections to contribute to oncogenic processes in several tumors, including oral, gastric, and colorectal cancer.^17,18,19^ In addition, esophageal candidiasis has been noted as potential driver of ESCC risk in patients with autoimmune polyendocrinopathy-candidiasis-ectodermal dystrophy.^20,21,22^

Esophageal candidiasis is also observed in patients with achalasia, but how frequently it occurs and to what extent this contributes to cancer-associated risk in these patients remain uninvestigated. Despite potential therapeutic and prognostic implications of concurrent esophageal *Candida* infection in achalasia, a comprehensive literature search yields very little structural data on candidiasis as a comorbidity of achalasia. Thus, this retrospective cohort study investigated the prevalence of esophageal *Candida* infection in patients with achalasia and assessed its association with EC development.

## Methods

### Study Populations

For this retrospective cohort study, individuals who were diagnosed with achalasia at the Erasmus University Medical Center or who were referred to the tertiary hospital for treatment and monitoring between January 1, 1980, and May 31, 2024, were identified through electronic database search. Given the long duration of this study, we accounted for the changes in the diagnosis criteria over time. Thus, the diagnosis of achalasia was consistently based on clinical symptoms, barium esophagram results, esophageal manometry, and upper endoscopy findings (eTable 1 in Supplement 1). Patients with incomplete diagnostic information for achalasia, lack of prior data before cancer diagnosis, or esophageal fungal infection that could not be confirmed as *Candida* species were excluded from the study. Written informed consent was obtained from patients who were still being monitored at the center at the time of the study. Patients who were lost to follow-up or deceased were excluded per the institutional review board protocol approved by the institutional ethics committee of the Erasmus University Medical Center. The study adhered to the Strengthening the Reporting of Observational Studies in Epidemiology (STROBE) reporting guideline.

For the analysis of the association between esophageal *Candida* infection and the development of EC, 1 patient for whom the time of diagnosis could not be accurately established, as well as 26 patients with achalasia but with only a single endoscopy report, were excluded. As these patients lacked long-term follow-up, their inclusion could lead to an underestimation of potential risks or incorrect associations.

### Primary Outcomes, Exposures, and Covariates

The primary outcomes included the prevalence of esophageal *Candida* infection and its association with the EC risk in patients with achalasia. The exposure was defined as 1 or more *Candida* infections during follow-up. The outcome was the progression of achalasia to EC, and covariates in the analysis included age at diagnosis and sex.

The primary study outcome, EC including high-grade dysplasia, was diagnosed by experienced gastroenterologists and pathologists based on pathological findings and imaging studies. Tumor subtypes, either esophageal adenocarcinoma or ESCC, were determined based on pathology findings. The follow-up period was defined as the time from first patient visit to Erasmus University Medical Center (baseline) to the occurrence of EC or the date of the last surveillance endoscopy at this center.

Data on esophageal *Candida* infection, including information from the medical records, endoscopy reports, pathology reports, and methods of detection and treatment, were systematically retrieved from electronic patient files and documented (eTable 1 in Supplement 1). Diagnosis of esophageal *Candida* infection was defined as clear white mucosal plaque–like lesions as determined by upper endoscopy findings and confirmed by histologic examination or periodic acid-Schiff staining showing the presence of yeast or hyphae invading mucosal cells. A comprehensive review and assessment of all patients’ clinical data was performed to identify the potential covariates, including baseline data (sex and age at diagnosis), the number of pneumatic dilation treatments, and history of esophageal myotomy.

### Statistical Analysis

Data were analyzed from August 1 to October 31, 2024. Descriptive statistics were used to describe characteristics of the entire cohort and the subcohort for cancer risk analysis. Nonnormally distributed continuous variables were reported as medians with IQRs, and categorical variables were presented as percentages. The Kaplan-Meier method was used to estimate the risk of esophageal *Candida* infection and the development EC in the entire cohort of patients with achalasia. Hazard ratios (HRs) and 95% CIs were calculated using Cox proportional hazards regression models to assess the associations between esophageal *Candida* infection, age at diagnosis, sex, and EC, with the status of esophageal *Candida* infection defined as a time-varying covariate. Additional multivariable Cox proportional hazards regression models were performed to obtain the adjusted hazard ratio (AHR) for esophageal *Candida* infection, adjusted for sex, and age at diagnosis. The same tests were repeated to investigate the association between esophageal *Candida* infection and ESCC risk. An extended standard Kaplan-Meier method was used to visualize the cumulative incidence of EC among patients with achalasia, stratified by the time-varying indicator of esophageal *Candida* infection. All statistical analyses were performed using SPSS, version 23 (SPSS Inc), and R, version 4.2.2 (R Project for Statistical Computing). A 2-tailed *P* < .05 was considered statistically significant.

## Results

### General Characteristics of the Study Population

**Figure 1.**
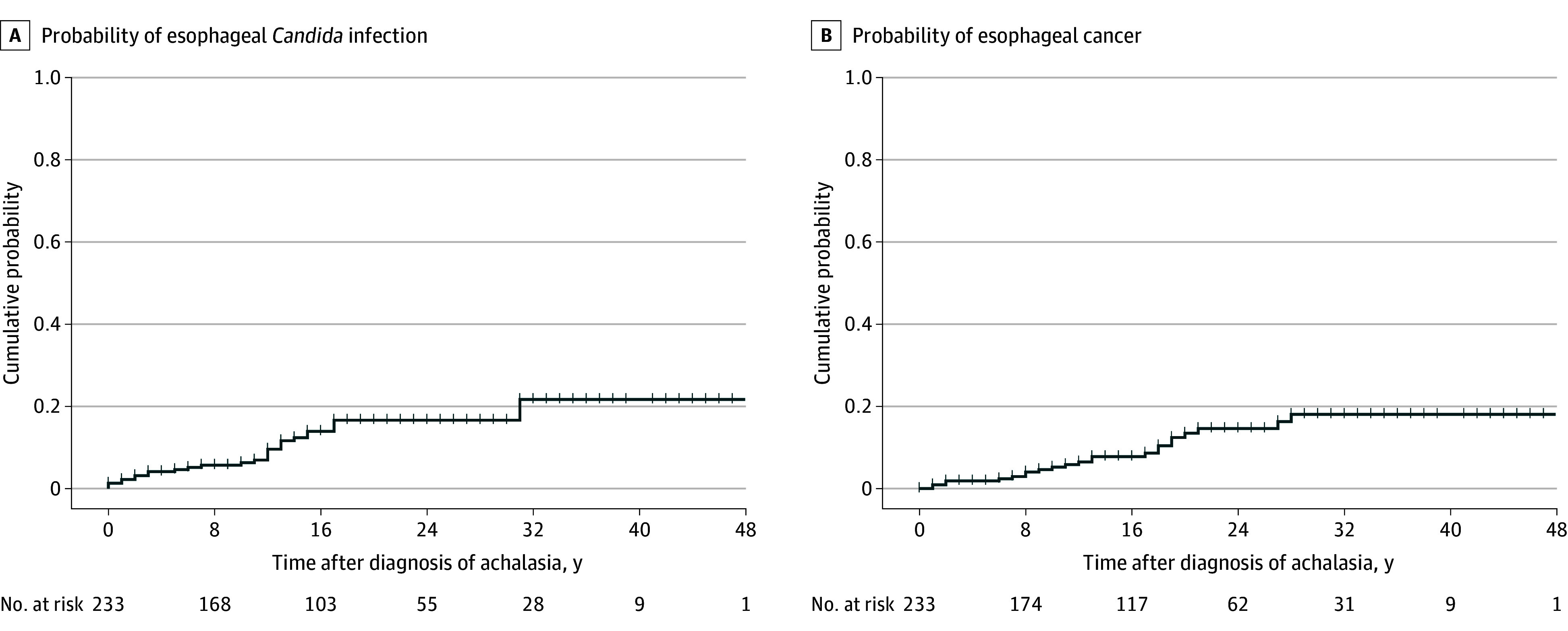
Kaplan-Meier Curves Illustrating Cumulative Probability of Esophageal *Candida* Infection and Esophageal Cancer Among All Patients With Achalasia Since Achalasia Diagnosis

Between 1980 and 2024, 234 patients with achalasia (median [IQR] age at diagnosis, 45 [32-63] years; 117 [50%] female and 117 [50%] male) who received diagnosis, treatment or monitoring at the Erasmus University Medical Center were included in this study. Demographic and clinical characteristics for the entire cohort are listed in Table 1. Among 234 patients, 190 (81%) were diagnosed with achalasia before 2000, 42 (18%) between 2000 and 2012, and only 1 patient (1%) was diagnosed with achalasia after 2012. The median (IQR) disease duration was 16 (8-24) years. A total of 227 patients (97%) received pneumatic dilation, with 88 patients (38%) receiving more than 1 dilation treatment. In addition to the pneumatic dilation, 30 patients (13%) underwent surgical myotomy as part of their treatment. During follow-up (median [IQR] duration, 13 [4-22] years), 29 patients (12%) experienced at least 1 esophageal *Candida* infection. As show in Figure 1A, esophageal *Candida* infection occurred primarily around the first 2 decades after diagnosis, with the cumulative risk of *Candida* infection reaching approximately 20% by 30 years after diagnosis. Esophageal cancer progression was observed in 24 patients (10%), of whom 19 (79%) developed ESCC. Figure 1B presents the Kaplan-Meier plot for the cumulative EC risk among patients with achalasia after diagnosis. The risk of developing EC increased gradually over time, with a notable rise observed approximately 10 to 20 years after the diagnosis of achalasia. A detailed timeline of achalasia-associated treatment, esophageal *Candida* infection, and disease progression for each patient who experienced esophageal candidiasis is shown in the eFigure in Supplement 1.

**Table 1.** Characteristics of the Total Study Population

Characteristic	Participants, No. (%) (N = 234)
Age at achalasia diagnosis, median (IQR), y	45 (32-63)
Sex	
Female	117 (50)
Male	117 (50)
Year of diagnosis	
<2000	190 (81)
2000-2012	42 (18)
>2012	1 (1)
Follow-up duration, median (IQR), y	13 (4-21)
Disease duration median (IQR), y	16 (8-24)
Pneumatic dilation	
1 time	139 (59)
2-3 times	79 (34)
>3 times	9 (4)
Myotomy treatment	
None	200 (86)
Heller	27 (12)
POEM	3 (1)
*Candida* infection	
With infection	29 (12)
Without infection	205 (88)
Esophageal cancer	
No progression	210 (90)
Unknow cancer type	2 (1)
EAC	3 (1)
ESCC	19 (8)

Abbreviations: EAC, esophageal adenocarcinoma; ESCC, esophageal squamous cell carcinoma; POEM, peroral endoscopic myotomy.

### Association Between Esophageal *Candida* Infection and EC in Achalasia

**Figure 2.**
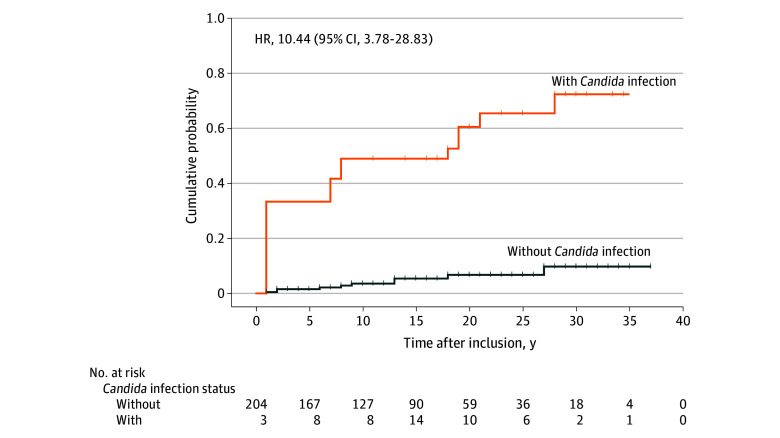
Extended Standard Kaplan-Meier Curves Illustrating Cumulative Incidence of Esophageal Cancer Among Patients With Achalasia, Stratified by Time-Varying Esophageal *Candida* Infection Status

In total, 207 patients (median [IQR] age at diagnosis, 43 [32-60] years; 104 [50%] female and 103 [50%] male) were included to investigate the association between esophageal *Candida* infection and the overall EC risk. The median (IQR) follow-up duration for this subgroup was 16 (9-26) years. The association between esophageal candidiasis and EC was investigated using Cox proportional hazards regression models, with esophageal *Candida* infection as a time-varying covariate. *Candida* infection was associated with increased risk of EC incidence (crude univariate HR, 10.44 [95% CI, 3.78-28.83]; *P* < .001) (Table 2). Figure 2 shows the cumulative incidence of EC among patients with achalasia stratified by time-varying esophageal *Candida* infection status. Additionally, older age at time of diagnosis (HR, 1.06 [95% CI, 1.03-1.10]; *P* < .001 and multivariate AHR, 1.06 [95% CI, 1.03-1.10]; *P* < .001) and male sex (HR, 3.06 [95% CI, 1.00-9.31]; *P* = .049; AHR, 3.34 [95% CI, 1.08-10.36]; *P* = .04) were also associated with increased risk of developing EC. After adjusting for age at diagnosis and male sex, esophageal *Candida* infection remained associated with increased risk of developing EC (AHR, 8.24 [95% CI, 2.97-22.89]; *P* < .001).

**Table 2.** Hazard Ratios for Esophageal Malignant Progression in Patients With Achalasia Based on Esophageal *Candida* Infection and Other Variables

Variable	Univariable analysis		Multivariable analysis	
	Crude HR (95% CI)	*P* value	Adjusted HR (95% CI)	*P* value
Esophageal cancer				
Age at diagnosis	1.06 (1.03-1.10)	<.001	1.06 (1.03-1.10)	<.001
Male sex	3.06 (1.00-9.31)	.049	3.34 (1.08-10.36)	.04
*Candida* infection	10.44 (3.78-28.83)	<.001	8.24 (2.97-22.89)	<.001
ESCC				
Age at diagnosis	1.07 (1.03-1.11)	<.001	1.07 (1.03-1.11)	<.001
Male sex	3.54 (1.00-12.54)	.05	3.98 (1.10-14.38)	.04
*Candida* infection	16.90 (5.45-52.34)	<.001	13.59 (4.34-42.51)	<.001

Abbreviations: ESCC, esophageal squamous cell carcinoma; HR, hazard ratio.

Achalasia is commonly associated with ESCC, while esophageal adenocarcinoma development in patients may arise as a consequence of a secondary development of Barrett esophagus (itself a risk factor for esophageal adenocarcinoma) due to gastric reflux induced by dilation treatments. Indeed, all 3 patients with achalasia who developed esophageal adenocarcinoma also had Barrett esophagus. We therefore performed a subanalysis including only patients who developed ESCC. This analysis showed that older age at diagnosis (HR, 1.07 [95% CI, 1.03-1.11]; *P* < .001) and male sex (HR, 3.54 [95% CI, 1.00-12.54]; *P* = .05) were also associated with higher risk of ESCC, and that patients who had an esophageal *Candida* infection showed a higher risk of progression to ESCC (HR, 16.90 [95% CI, 5.45-52.34]; *P* < .001). After adjusting for age at diagnosis and male sex, patients with achalasia and prior esophageal *Candida* infection demonstrated an approximately 13-fold increased risk of developing ESCC (AHR, 13.59 [95% CI, 4.34-42.51]; *P* < .001).

Given that patient inclusion in our cohort started in the early 1980s and that management of disease may have changed over time, we also included the year of diagnosis as a covariate in the Cox proportional hazards regression model. The results of this analysis indicated that the year of achalasia diagnosis was not associated with EC progression (HR, 1.02 [95% CI, 0.02-0.77]; *P* = .44).

## Discussion

In this retrospective cohort study of 234 patients with achalasia, we found that the prevalence of esophageal *Candida* infection was 12%. Risk of developing EC was significantly increased in patients with achalasia who had a history of esophageal *Candida* infection.

*Candida* is a genus of yeast that is considered to be part of the normal microbiota in the human gastrointestinal and genitourinary tracts. However, this microbe has the potential to invade and cause severe infections when there is an imbalance in the ecological niche, the mucosal barrier is disrupted, or the immune system of the host is compromised. The first description of *Candida* infection in obstructive disease, including achalasia, dates from 1981, at which time the authors suggested that esophageal stasis of any cause can lead to esophageal candidiasis.^23^ Some studies suggest that esophageal candidiasis is frequent enough in patients with achalasia to warrant preventative fluconazole treatment of patients prior to surgical treatment.^24^ Prior to our cohort analysis, we conducted a comprehensive literature review to investigate the prevalence of esophageal *Candida* infection reported in earlier studies about achalasia (eMethods in Supplement 1). However, this search did not identify any studies directly focused on this topic (eTable 2 in Supplement 1).

In our retrospective cohort study, encompassing a relatively large number of patients with achalasia and a long follow-up period, 12% of patients with achalasia experienced at least 1 episode of esophageal *Candida* infection. The prevalence of esophageal *Candida* infection in achalasia observed in our study falls within the range reported in the literature (eTable 2 in Supplement 1), but is much higher than that reported in the general population (0.32%-5.2%).^25,26^ While some studies reported esophageal candidiasis as an adverse event during follow-up after surgical treatment (peroral endoscopic myotomy, Heller myotomy),^27,28,29,30^ considering that the investigation of this adverse event was at least 1 year after the surgical treatment, it is plausible that this event represents a secondary infection. This speculation is further supported by the observation in the present cohort that esophageal *Candida* infection was observed up to 12 years after myotomy in some cases, and that esophageal *Candida* infection was common in patients with achalasia even without peroral endoscopic myotomy or Heller myotomy treatment (eFigure in Supplement 1). Our study also showed that 97% of patients with esophageal *Candida* infection previously received pneumatic dilation. Together, these findings emphasize the high prevalence of esophageal *Candida* infection in patients with achalasia and that common treatments such as pneumatic dilation do not reduce the possibility of *Candida* infection.

Increasing evidence suggests that the microbiota, including fungal infections, may contribute to cancer risk.^13^ Because *Candida* species are the most common human fungal pathogen, the association between candidiasis and cancer has been noticed for decades. Studies have shown *Candida* infection to be present in 27% of patients with EC,^31^ with up to 57% of patients with EC having positive fungal culture results.^32^ The preferred hypothesis here is that candidiasis can develop secondary to cancer due to impaired antifungal host immunity or mucosal damage. Nevertheless, studies have shown that *Candida* infection is also associated with the progression of oral mucosal epithelial dysplasia^33,34^ and is linked to increased oral tumor–associated macrophages via the interleukin (IL)-17A/IL-17RA pathway.^35^ Increased *Candida albicans* burden can also promote the development of colon cancer by inducing glycolysis and IL-7 secretion.^19^ Collectively, these findings suggest *Candida* infection may be associated with the process of carcinogenesis through quorum sensing–induced changes in virulence factor expression.^36^ However, to our knowledge, no subsequent studies have further investigated or validated this hypothesis, and it is unknown whether *Candida* infection plays a similar role in the development of cancerous conditions in achalasia.

Retrospective analysis of patients with achalasia being monitored at the Erasmus University Medical Center indicates that overall, 10.2% of these patients develop EC, which is close to the reported range for the incidence of ESCC in achalasia of 0.4% to 9.2%.^37^ Studies have shown that surveillance endoscopy can significantly reduce EC-related mortality by facilitating the early detection of the EC.^38^ However, effective surveillance in this population is challenging due to the prolonged disease duration and the lack of factors to accurately stratify individuals at real risk. In the present study, incorporating esophageal *Candida* infection as a time-varying covariate revealed that patients with achalasia and esophageal *Candida* infection had an 8.2-fold increased risk of developing EC and a 13.6-fold increased risk of ESCC. In addition, male sex and older age at achalasia diagnosis were also associated with an increased risk for EC and ESCC. These findings underscore the necessity of long-term follow-up of patients with achalasia, particularly for male patients diagnosed at an older age in whom esophageal *Candida* infection has been observed.

### Limitations

We acknowledge several limitations to our study. First, this is a single-center study that included many patients who were diagnosed with achalasia between 1974 and 2016. As achalasia subtypes were only distinguished in later guidelines, information on the type of achalasia was not available for most patients. While the proposed mechanism by which *Candida* infection is involved in malignant progression (alteration of the immune environment within the esophageal mucosa) is a process in which the type of achalasia is not critically relevant, it is conceivable that patients have different susceptibility to *Candida* infection during different stages of disease. Second, known risk factors for ESCC, including alcohol intake, smoking, and nitrosamine consumption, were not included in this study as we were unable to retrieve these data for all patients retrospectively. However, our results are consistent with a former study showing that ESCC is more frequently reported in males, likely due to increased smoking and alcohol intake.^10^ More important, the underlying mechanisms contributing to the risk or EC development by alcohol intake and nitrosamine consumption are still unknown. Studies have suggested that *Candida* species can metabolize ethanol into acetaldehyde, leading to an increase of local acetaldehyde in the esophagus, thus enhancing the carcinogenic effect of alcohol.^39,40^ Additionally, the catalytic activity of *Candida* species facilitates the production of carcinogenic nitrosamines, such as *N*-nitrosobenzylmethylamine, from their precursors.^41^ It is tempting to speculate that esophageal *Candida* species may act as an intermediate factor linking these risk factors to the development of EC. Last, despite our describing one of the largest longitudinal achalasia cohorts to date, our cohort included relatively few cancer cases, allowing for inclusion of a limited number of covariates in our risk factor analysis. Whether *Candida* infection directly modulates esophageal carcinogenesis needs to be evaluated in larger studies.

## Conclusions

This retrospective cohort study showed a high prevalence of esophageal *Candida* infection among patients with achalasia and found that prior *Candida* infection was associated with an increased EC risk. Findings from our study emphasize the necessity of improved reporting of esophageal candidiasis during the surveillance of achalasia, and patients diagnosed with esophageal *Candida* infection should be considered for regular surveillance endoscopy for early detection of EC.

## Notes

Supplement 1. **eTable 1.** Overview of Diagnostic and Therapeutic Approaches for Achalasia and *Candida* Infection in this Cohort **eMethods.** Search Strategies for Literature on the Prevalence of *Candida* Infection in Patients With Achalasia **eTable 2.** Cohort Studies Identified in Systematic Review with Mention of Achalasia With Concurrent Esophageal Candidiasis **eFigure.** Sequential Timeline of Treatments, Esophageal *Candida* Infection and Disease Progression in Achalasia Patients
eReferences.

Supplement 2.
Data Sharing Statement
