# Supplementary material for: Esophageal Candida Infection and Esophageal Cancer Risk in Patients With Achalasia
Source: JAMA Netw Open. 2025 Jan 14;8(1):e2454685. doi: 10.1001/jamanetworkopen.2024.54685 (PMC11733698; doi:10.1001/jamanetworkopen.2024.54685)
Supplement: Supplement 1. — eTable 1. Overview of Diagnostic and Therapeutic Approaches for Achalasia and Candida Infection in this Cohort eMethods. Search Strategies for Literature on the Prevalence of Candida Infection in Patients With Achalasia eTable 2. Cohort Studies Identified in Systematic Review with Mention of Achalasia With Concurrent Esophageal Candidiasis eFigure. Sequential Timeline of Treatments, Esophageal Candida Infection and Disease Progression in Achalasia Patients eReferences. [file jamanetwopen-e2454685-s001.pdf]

# Supplementary Online Content

Guo X, Lam SY, Janmaat VT, et al. Esophageal *Candida* infection and esophageal cancer risk in patients with achalasia. *JAMA Netw Open*. 2025;8(1):e2454685.
doi:10.1001/jamanetworkopen.2024.54685

**eTable 1.** Overview of Diagnostic and Therapeutic Approaches for Achalasia and *Candida* Infection in this Cohort

**eMethods.** Search Strategies for Literature on the Prevalence of *Candida* Infection in Patients With Achalasia.

**eTable 2.** Cohort Studies Identified in Systematic Review with Mention of Achalasia With Concurrent Esophageal Candidiasis

**eFigure.** Sequential Timeline of Treatments, Esophageal *Candida* Infection and Disease Progression in Achalasia Patients

**eReferences.**

This supplementary material has been provided by the authors to give readers additional information about their work.

© 2025 Guo X et al. *JAMA Network Open*.

**eTable 1. Overview of Diagnostic and Therapeutic Approaches for Achalasia and *Candida* Infection in this Cohort**

| Characteristic | Entire Cohort, No. (%) (n=234) |
|---|---|
| Diagnosis Hospital | |
| EMC | 121 (51.7) |
| Other | 113 (48.3) |
| Diagnosis of Achalasia | |
| Upper Endoscopy | 221 (94.4) |
| Barium esophagram | 221 (94.4) |
| Esophageal Manometry | 212 (90.6) |
| Diagnosis of *Candida* infection | |
| Endoscopy | 3 (10.2) |
| Histological examination | 26 (89.7) |
| Periodic acid-Schiff (PAS) staining | 2 (6.9) |
| Anti-fungal Treatment | |
| Unknown | 13 (44.8) |
| Nystatin | 7 (24.1) |
| Fluconazole | 8 (27.6) |
| Fungizone | 1 (3.4) |

© 2025 Guo X et al. *JAMA Network Open*.

**eMethods. Search Strategies for Literature on the Prevalence of *Candida* Infection in Patients With Achalasia**

We systematically searched Embase database, Medline Ovid, Web of science, Cochrane CENTRAL and Google scholar to identify the studies providing information on the prevalence of *Candida* or fungal infection in achalasia. Case studies conference abstracts and studies not in English language were excluded

| Database searched | via | Years of coverage | Records | Records after duplicates removed |
|---|---|---|---|---|
| Embase | Embase.com | 1971 - Present | 167 | 165 |
| Medline ALL | Ovid | 1946 - Present | 41 | 15 |
| Web of Science Core Collection[a] | Web of Knowledge | 1975 - Present | 24 | 2 |
| Cochrane Central Register of Controlled Trials | Wiley | 1992 - Present | 2 | 0 |
| *Other sources: Google Scholar* | | | 50 | 32 |
| **Total** | | | **284** | **214** |

[a]Science Citation Index Expanded (1975-present); Social Sciences Citation Index (1975-present); Arts & Humanities Citation Index (1975-present); Conference Proceedings Citation Index- Science (1990-present); Conference Proceedings Citation Index- Social Science & Humanities (1990-present); Emerging Sources Citation Index (2015-present)

**Embase**

('esophagus achalasia'/de OR (achalasia* OR cardiospas* OR ((cardia* OR cardio*) NEAR/3 (spas*))):ab,ti,kw) **AND** ('mycosis'/exp OR (mycos* OR mycot* OR candidias* OR monilias* OR chytridiomycos* OR dermatomycos* OR eumycetoma* OR geotrichos* OR hyalohyphomycos* OR microsporidios* OR mycotoxicos* OR oomycos* OR otomycos* OR phaeohyphomycos* OR pneumocyst* OR zygomycos* OR candida OR candidos* OR aspergill* OR neuroaspergill* OR

© 2025 Guo X et al. *JAMA Network Open*.

coccidioidomycos* OR cryptococc* OR blastomycos*OR chromoblastomycos* OR hyalohyphomycos* OR lobomycos* OR mycetom* OR sporotrichos* OR tinea OR suppurative-uveitis* OR histoplasmos* OR fungemia OR encephalitozoonos* OR paracoccidioidomycosis OR piedra OR trichosporonos* OR mucormycos* OR ((fungal* OR fungus*) NEAR/3 (infect* OR diseas* OR invasion* OR endocardit* OR meningit* OR sinusit* OR ball))):ab,ti,kw)

**Medline**

(Esophageal Achalasia/ OR (achalasia* OR cardiospas* OR ((cardia* OR cardio*) ADJ3 (spas*))).ab,ti,kf.) AND (exp Mycoses/ OR (mycos* OR mycot* OR candidias* OR monilias* OR chytridiomycos* OR dermatomycos* OR eumycetoma* OR geotrichos* OR hyalohyphomycos* OR microsporidios* OR mycotoxicos* OR oomycos* OR otomycos* OR phaeohyphomycos* OR pneumocyst* OR zygomycos* OR candida OR candidos* OR aspergill* OR neuroaspergill* OR coccidioidomycos* OR cryptococc* OR blastomycos*OR chromoblastomycos* OR hyalohyphomycos* OR lobomycos* OR mycetom* OR sporotrichos* OR tinea OR suppurative-uveitis* OR histoplasmos* OR fungemia OR encephalitozoonos* OR paracoccidioidomycosis OR piedra OR trichosporonos* OR mucormycos* OR ((fungal* OR fungus*) ADJ3 (infect* OR diseas* OR invasion* OR endocardit* OR meningit* OR sinusit* OR ball))).ab,ti,kf.)

**Cochrane**

((achalasia* OR cardiospas* OR ((cardia* OR cardio*) NEAR/3 (spas*))):ab,ti,kw) **AND** ((mycos* OR mycot* OR candidias* OR monilias* OR chytridiomycos* OR dermatomycos* OR eumycetoma* OR geotrichos* OR hyalohyphomycos* OR microsporidios* OR mycotoxicos* OR

© 2025 Guo X et al. *JAMA Network Open*.

oomycos* OR otomycos* OR phaeohyphomycos* OR pneumocyst* OR zygomycos* OR candida OR candidos* OR aspergill* OR neuroaspergill* OR coccidioidomycos* OR cryptococc* OR blastomycos*OR chromoblastomycos* OR hyalohyphomycos* OR lobomycos* OR mycetom* OR sporotrichos* OR tinea OR suppurative-uveitis* OR histoplasmos* OR fungemia OR encephalitozoonos* OR paracoccidioidomycosis OR piedra OR trichosporonos* OR mucormycos* OR ((fungal* OR fungus*) NEAR/3 (infect* OR diseas* OR invasion* OR endocardit* OR meningit* OR sinusit* OR ball))):ab,ti,kw)

**Web of Science**

TS=(((achalasia* OR cardiospas* OR ((cardia* OR cardio*) ADJ3 (spas*)))) AND ((mycos* OR mycot* OR candidias* OR monilias* OR chytridiomycos* OR dermatomycos* OR eumycetoma* OR geotrichos* OR hyalohyphomycos* OR microsporidios* OR mycotoxicos* OR oomycos* OR otomycos* OR phaeohyphomycos* OR pneumocyst* OR zygomycos* OR candida OR candidos* OR aspergill* OR neuroaspergill* OR coccidioidomycos* OR cryptococc* OR blastomycos*OR chromoblastomycos* OR hyalohyphomycos* OR lobomycos* OR mycetom* OR sporotrichos* OR tinea OR suppurative-uveitis* OR histoplasmos* OR fungemia OR encephalitozoonos* OR paracoccidioidomycosis OR piedra OR trichosporonos* OR mucormycos* OR ((fungal* OR fungus*) ADJ3 (infect* OR diseas* OR invasion* OR endocardit* OR meningit* OR sinusit* OR ball)))))

**Google Scholar – 50 refs**

© 2025 Guo X et al. *JAMA Network Open*.

achalasia mycosis|mycoses|"fungal|fungus

infection|disease|invasion|endocarditis|meningitis|sinusitis"|candidiasis

© 2025 Guo X et al. *JAMA Network Open*.

**eTable 2. Cohort Studies Identified in Systematic Review With Mention of Achalasia with Concurrent Esophageal Candidiasis.**

| Author / Years | Country | # achalasia pt | Diagnostic method | Fungal infection N (%) | Method of detection | Primary/Secondary | Primary reason for study | Type of study |
|---|---|---|---|---|---|---|---|---|
| Ponds et al, (2019)[1] | Netherlands, Germany, Italy, Hong Kong, US | 130 | HRM | 3 (2.25%) | Unreported | Post-treatment | Compare the outcome of POEM vs. PD | Multicenter randomized clinical trial |
| Sonnenberg et al, (1993)[2] | US | 21376 | ICD code | 44 (0.21%) | ICD code | Unreported | Epidemiology and comorbidities of achalasia | National data registry study |
| Andolfi et al, (2016)[3] | US, Italy | 524 | HRM, ES | 8% | Endoscopy | Pre-treatment | Misdiagnosis of Achalasia as GERD | Multicenter retrospective chart review |
| Cools-Lartigue et al, (2013)[4] | Canada | 50 | EGD | 7 (14%) | Histology, endoscopy | Pre-treatment | Incidence eosinophilic infiltration in achalasia | Single center prospective chart study |
| Kolb et al, (2020)[5] | US | 6 | HRM, ES | 1 (16.7%) | Endoscopy | Post-treatment | Safety of POEM in bariatric surgery patients | Single center prospective cohort chart review |
| Gossage et al, (2014)[6] | Australia | 68 | Unreported | 5 (7.4%) | Histology | Post-treatment | Outcomes of cardiomyopathy | Multicenter prospective cohort study |
| Tebaibia et al, (2016)[7,8] | Algeria | 1256 | EGD, EM | 75 (6%) | Endoscopy | Unreported | Epidemiology and incidence of achalasia | Single center prospective cohort chart review |
| Scott et al, (1982)[8] | UK | 4 | Unreported | 1 (25%) | Histology | Unreported | Epidemiology and incidence of esophageal candidiasis | Single center prospective cohort chart review |
| Kjellin et al, (2005)[9] | Sweden | 26 | EM | 3 (11.5%) | Endoscopy | Pre-treatment | Histological features of achalasia | Single center prospective evaluation |
| Lee et al, (2021)[10] | Korea | 22 | EM, barium | 3 (13.6%) | Endoscopy | Unreported | Histological features of achalasia | Single center retrospective chart review |
| Lindenmann et al,(2005)[11] | Austria | 40 | Unreported | 1 (2.5%) | Endoscopy | Post-treatment | Outcome of transthoracic Heller | Single center retrospective chart study |
| Eckardt et al, (2008)[12] | Germany | 253 | EM | 5 (2%) | Unreported | Post-treatment | Morbidity of achalasia | Single center prospective cohort study |

PD: Pneumatic dilation; HRM: High resolution manometry; ICD: 9th classification if international classification of diagnoses; ES: Eckhardt score; GERD: gastroesophageal reflux disease; IEGD: esophagogastroduodenoscopy; EM: esophageal manometry.

© 2025 Guo X et al. *JAMA Network Open*.

**eFigure. Sequential Timeline of Treatments, Esophageal *Candida* infection and Disease Progression in Achalasia Patients**

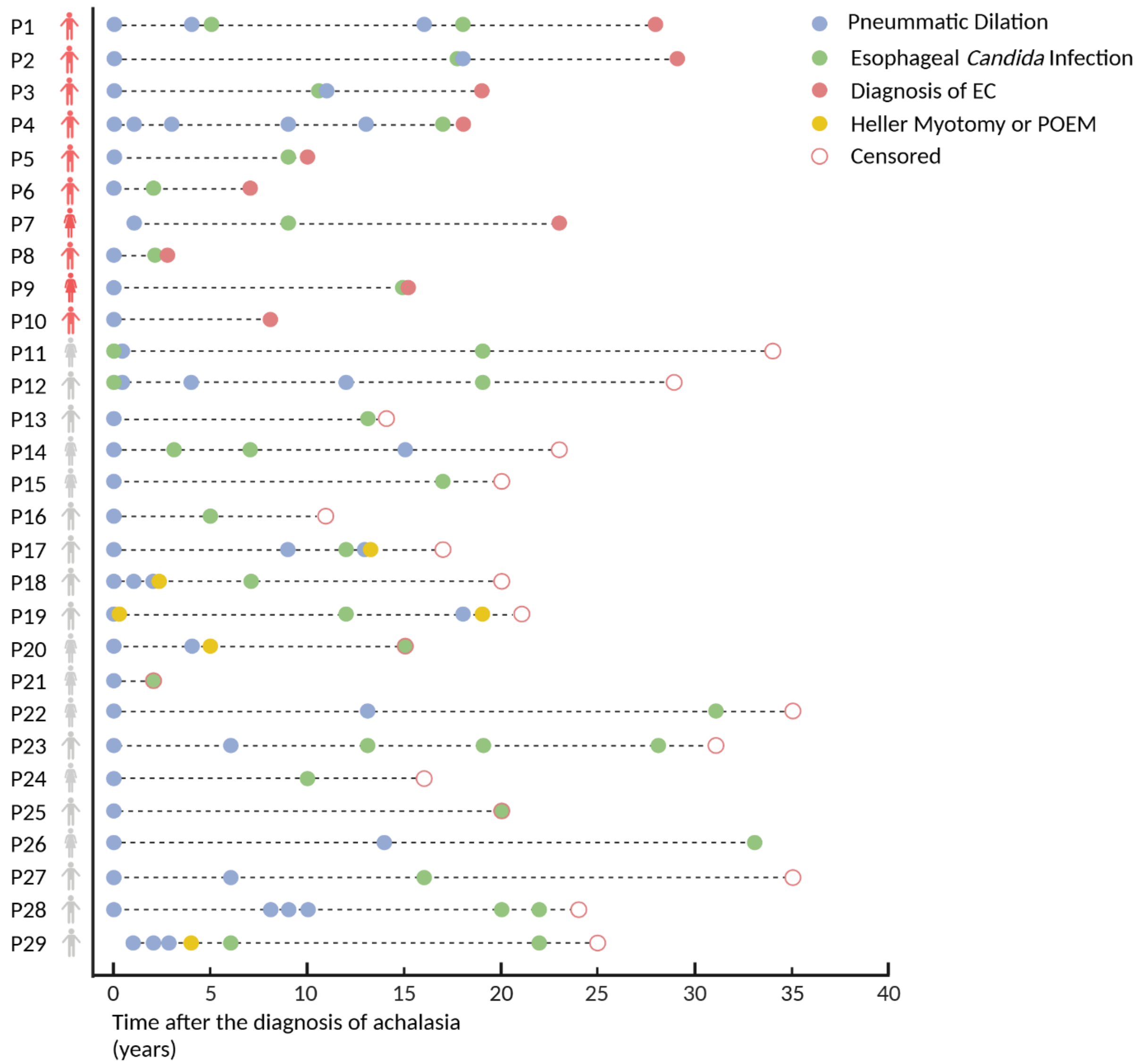


Timeline for all patients diagnosed with esophageal *Candida* infection during the course of follow-up. Gender is indicated for each patient, as is the sequence of events with regards to treatment (Pneumatic dilation in blue, Heller myotomy or POEM in yellow), *Candida* diagnosis (green), cancer development (pink) and censure (open pink circle). Figure was produced with the use of BioRender.

© 2025 Guo X et al. *JAMA Network Open*.

**eReferences**


1. Ponds FA, Fockens P, Lei A, et al. Effect of Peroral Endoscopic Myotomy vs Pneumatic Dilation on Symptom Severity and Treatment Outcomes among Treatment-Naive Patients with Achalasia: A Randomized Clinical Trial. JAMA 2019;322:134-144.
2. Sonnenberg A, Massey BT, McCarty DJ. Epidemiology of hospitalization for achalasia in the United States. Digestive diseases and … 1993.
3. Andolfi C, Bonavina L, Kavitt RT, et al. Importance of esophageal manometry and pH monitoring in the evaluation of patients with refractory gastroesophageal reflux disease: A multicenter study. J Laparoendosc Adv Surg Techn 2016;26:548-550.
4. Cools-Lartigue J, Chang SY, McKendy K, et al. Pattern of esophageal eosinophilic infiltration in patients with achalasia and response to Heller myotomy and Dor fundoplication. Dis Esophagus 2013;26:766-775.
5. Kolb JM, Jonas D, Funari MP, et al. Efficacy and safety of peroral endoscopic myotomy after prior sleeve gastrectomy and gastric bypass surgery. World J Gastrointest Endosc 2020;12:532-541.
6. Gossage JA, Devitt PG, Watson DI, et al. Surveillance endoscopy at five or more years after cardiomyotomy for achalasia. Ann Surg 2014;259:464-468.
7. Tebaibia A, Boudjella MA, Boutarene D, et al. Incidence, clinical features and para-clinical findings of achalasia in Algeria: Experience of 25 years. World J Gastroenterol 2016;22:8615-8623.
8. Scott BB, Jenkins D. Gastro-oesophageal candidiasis. Gut 1982.
9. Kjellin AP, Öst ÅEI, Pope Ii CE. Histology of esophageal mucosa from patients with achalasia. Dis Esophagus 2005;18:257-261.
10. Lee BE, Kim GH, Shin N, et al. Histopathological analysis of esophageal mucosa in patients with achalasia. Gut Liver 2021;15:713-722.
11. Lindenmann J, Maier A, Eherer A, et al. The incidence of gastroesophageal reflux after transthoracic esophagocardio-myotomy without fundoplication: A long term follow-up. Eur J Cardio-thorac Surg 2005;27:357-360.
12. Eckardt VF, Hoischen T, Bernhard G. Life expectancy, complications, and causes of death in patients with achalasia: Results of a 33-year follow-up investigation. Eur J Gastroenterol Hepatol 2008;20:956-960.


© 2025 Guo X et al. *JAMA Network Open*.
