# Supplementary material for: Esophageal Candida Infection and Esophageal Cancer Risk in Patients With Achalasia
Source: JAMA Netw Open. 2025 Jan 14;8(1):e2454685. doi: 10.1001/jamanetworkopen.2024.54685 (PMC11733698; doi:10.1001/jamanetworkopen.2024.54685)
Supplement: Supplement 2. — Data Sharing Statement [file jamanetwopen-e2454685-s002.pdf]

Data Sharing Statement

Guo. Esophageal Candida Infection and Esophageal Cancer Risk in Patients with Achalasia. *JAMA Netw Open*. Published January 14, 2025. doi:10.1001/jamanetworkopen.2024.54685

Data
Data available: No
